# 19-Gauge Versus 22-Gauge Franseen Needles, Comparison of the Histological Diagnostic Capability of Endoscopic Ultrasound-Guided Fine-Needle Biopsy for Autoimmune Pancreatitis: A Multicenter Retrospective Cohort Study

**DOI:** 10.3390/diagnostics15121496

**Published:** 2025-06-12

**Authors:** Shota Iwata, Takuji Iwashita, Yosuke Ohashi, Akihiko Senju, Ryuichi Tezuka, Shinya Uemura, Kensaku Yoshida, Akinori Maruta, Yuhei Iwasa, Mitsuru Okuno, Keisuke Iwata, Tatsuhiko Miyazaki, Masahito Shimizu

**Affiliations:** 1 First Department of Internal Medicine, Gifu University Hospital, Gifu 501-1194, Japan; iwata.shota.y1@f.gifu-u.ac.jp (S.I.); yosuke-ohashi14@hotmail.com (Y.O.); senjuakihiko1989@gmail.com (A.S.); tez1101@gmail.com (R.T.); ueshin550621@gmail.com (S.U.); shimizu.masahito.j1@f.gifu-u.ac.jp (M.S.); 2 Department of Gastroenterology, Gifu Prefectural General Medical Center, Gifu 500-8717, Japan; kensakuyoshidaky@gmail.com (K.Y.); mrak5844@yahoo.co.jp (A.M.); 3 Department of Gastroenterology, Gifu Municipal Hospital, Gifu 500-8513, Japan; festinalenteyu@gmail.com (Y.I.); mkobdkl@yahoo.co.jp (M.O.); keisukeiwata827@gmail.com (K.I.); 4 Department of Pathology, Gifu University Hospital, Gifu 501-1194, Japan; miyazaki.tatsuhiko.z0@f.gifu-u.ac.jp

**Keywords:** autoimmune pancreatitis, 19-gauge, 22-gauge, endoscopic ultrasound-guided fine needle biopsy, Franseen needle

## Abstract

**Background/Objectives**: Endoscopic ultrasound-guided fine-needle biopsy (EUS-FNB) is a useful procedure for obtaining histological specimens. However, its utility in diagnosing autoimmune pancreatitis (AIP) has not yet been well studied. This study aimed to assess the diagnostic capability of EUS-FNB for AIP by comparing a 19-gauge Franseen needle (19FR) and a 22-gauge Franseen needle (22FR). **Methods**: This study included patients with a final diagnosis of AIP undergoing EUS-FNB for pancreatic lesions between January 2014 and February 2023. All patients underwent EUS-FNB with either 19FR or 22FR. Histological findings were evaluated according to the International Consensus Diagnostic Criteria (ICDC). The primary outcome was the diagnostic yield of Level 1 (≥3 ICDC items) or Level 2 (2 ICDC items). **Results**: The 19FR group included 31 patients, and the 22FR group included 36 patients. The Level 1 diagnostic rate was significantly higher in the 19FR group than in the 22FR group (90.3% vs. 61.1%, *p* = 0.010). No significant difference was observed in the Level 2 diagnostic rate. The 19FR group yielded significantly larger histological tissue samples than the 22FR group (median area: 9.19 mm^2^/session vs. 3.36 mm^2^/session, *p* < 0.001). The analysis demonstrated a positive correlation between tissue area and the number of histological diagnostic items obtained. **Conclusions**: EUS-FNB performed with the 19FR provided larger histological specimens and a higher histological diagnostic yield than the 22FR in the diagnosis of AIP. Obtaining a larger amount of tissue may facilitate a definitive diagnosis of AIP.

## 1. Introduction

Autoimmune pancreatitis (AIP) was first recognized as a distinct clinical entity by Yoshida et al. in 1995 as chronic pancreatitis with a favorable response to steroids, which is related to autoimmune mechanisms [1]. Since then, its epidemiology, diagnostic methods, treatment, and prognosis have been studied by many researchers. A variety of diagnostic criteria have been reported, but the basic criteria are based on elevated serum IgG4 levels, diffuse or localized pancreatic enlargement associated with narrowing of the main pancreatic duct on imaging studies, characteristic histological findings, and response to steroid therapy. Pathological examination is particularly important for differentiating AIP from other diseases, particularly pancreatic cancer, and for establishing a definitive diagnosis of AIP [2]. The histological findings of AIP are classified as lymphoplasmacytic sclerosing pancreatitis type 1 AIP, characterized by lymphoplasmacytic infiltration, storiform fibrosis, IgG4-positive plasma cell infiltration, and obliterative phlebitis [3,4]; and idiopathic duct-centric pancreatitis type 2 AIP, characterized by granulocytic epithelial lesions [5,6,7].

While pathological examination is important for the differential and definitive diagnosis of AIP, obtaining sufficient tissue for diagnosis can be challenging. Endoscopic ultrasound-guided fine-needle aspiration (EUS-FNA) has been reported to have a low diagnostic yield [5,8]. Therefore, the International Consensus Diagnostic Criteria (ICDC) developed in 2011 do not recommend EUS-FNA as a method for specimen collection; instead, core biopsy or resection is recommended [3]. In recent years, fine-needle biopsy (FNB) needles with unique tip shapes have been developed and reported to be useful for histological specimen collection during EUS-FNB [9,10,11,12,13,14,15,16,17,18,19]. However, there have been few reports on the usefulness of EUS-FNB in the diagnosis of AIP, and the selection of needle size and tip shape has scarcely been studied. In this study, we compared the histological diagnostic capability of EUS-FNB using a 19-gauge Franseen needle (19FR) and a 22-gauge Franseen needle (22FR) for pancreatic lesions in AIP.

## 2. Materials and Methods

### 2.1. Study Design

This multicenter, retrospective cohort study was performed at Gifu University Hospital, Gifu Prefectural General Medical Center, and Gifu Municipal Hospital. A database analysis was conducted, encompassing all EUS-FNA procedures between January 2014 and February 2023, to identify patients who fulfilled the following criteria. We included (1) patients who underwent EUS-FNB using a Franseen needle for a pancreatic lesion and (2) those with a final diagnosis of AIP. However, patients were excluded if (1) a 25FR was used, (2) multiple needle types were used, or (3) they underwent puncture from both the stomach and duodenum in a single session. AIP was diagnosed based on the ICDC, considering imaging features, serology, histopathology of the pancreas, presence of other organ involvement, and response to steroids. The final diagnosis was confirmed by follow-up of 6 months. This study was conducted in accordance with the Declaration of Helsinki. The study protocol was approved by the Institutional Review Board of each hospital (Gifu University Hospital: 2023-161).

### 2.2. EUS-FNB

All EUS-FNB procedures were performed by endoscopists with experience in EUS-related procedures using linear-array echoendoscopes (GF-UCT260, Olympus, Tokyo, Japan, or EG-740UT, Fujifilm, Tokyo, Japan). EUS-FNB was performed under conscious sedation using midazolam and pentazocine, with continuous monitoring of vital signs. An EUS device was inserted orally to visualize the pancreas. After confirming the absence of the main pancreatic duct and blood vessels in the puncture line using B-mode and color Doppler, the pancreatic lesion was punctured from the stomach or duodenum (bulb or second portion). A 19FR (Acquire, Boston Scientific Corporation, Marlborough, MA, USA; SonoTip TopGain, Medi-Globe, Achenmühle, Germany) and a 22FR (Acquire, Boston Scientific Corporation, Marlborough, MA, USA) were used. After stylet removal and application of 10 cc of negative pressure, the needle tip was moved back and forth within the lesion five to ten times. After the suction was released, the needle was withdrawn into the sheath, and the entire FNB system was removed. The stylet was then reinserted into the FNB needle, and the obtained specimens were expelled onto glass slides to check for the presence of white specimens. Macroscopic On-Site Evaluation was performed as previously described [20]. The collected specimens were separated into blood and whitish portions. The whitish portions were fixed in formalin for histological examination, while the remaining material was smeared onto glass slides and fixed with alcohol for cytological analysis.

### 2.3. Pathological Evaluations

**Figure 1:**
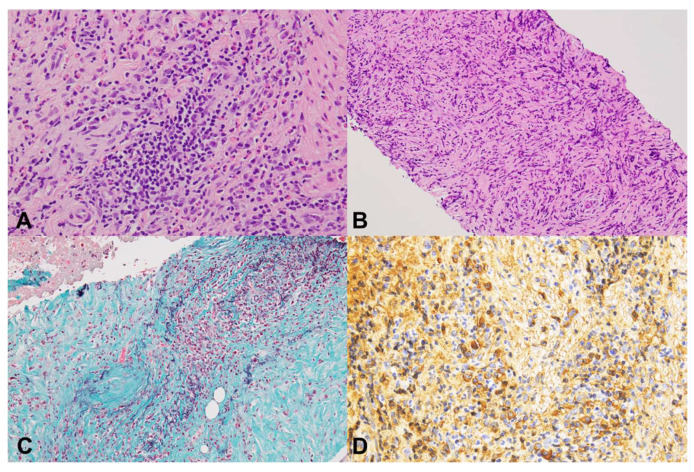
Histopathological findings of autoimmune pancreatitis (AIP) obtained with Franseen needles. (**A**) lymphoplasmacytic infiltration (H&E staining ×400). (**B**) storiform fibrosis (H&E staining ×200). (**C**) obliterative phlebitis (Elastica Masson staining ×200). (**D**) abundant IgG4-positive cells (>10/HPF) (IgG4 immunostaining ×400).

**Figure 2:**
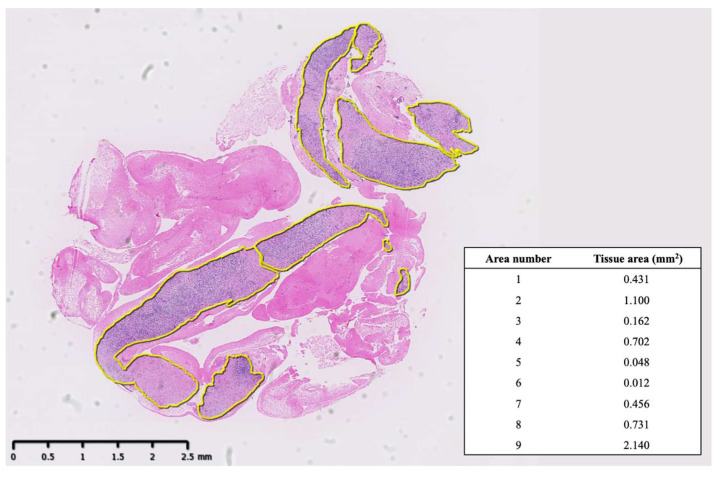
The evaluation of tissue area on histological examination. The total area of tissue samples was surrounded with a yellow line using imaging software (NDP.view 2.9.29).

Cytological and histological evaluations were conducted by a pathologist (T.M.) who was blinded to the type of puncture needle. Histological examination was performed using hematoxylin and eosin and IgG4 staining. When veins were small and difficult to evaluate, Elastica van Gieson (EVG) staining or Elastica-Masson staining was performed to assess obliterative phlebitis. According to the ICDC, the following four pathological features of AIP were evaluated: (1) lymphoplasmacytic infiltration, (2) storiform fibrosis, (3) obliterative phlebitis, and (4) abundant IgG4-positive cells (>10 cells per high-power field [HPF]) (Figure 1). Storiform fibrosis was defined as the presence of inflammatory cells, spindle-shaped cells, and collagen fibers arranged in a flowing pattern. To evaluate the tissue area on histological examination, the glass slides were converted into virtual slides using a virtual slide scanner (NanoZoomer; Hamamatsu Photonics, Hamamatsu City, Japan). A still image was captured from the virtual slide, and the total area of the tissue samples, excluding blood and mucus, was calculated using imaging software (NDP.view 2.9.29; Hamamatsu Photonics, Japan) (Figure 2).

### 2.4. Study Definitions

A histologically adequate specimen was defined as one that allowed sufficient evaluation of the tissue architecture to differentiate it from pancreatic cancer. The histopathological diagnosis of AIP was based on the ICDC. Level 1 diagnosis requires ≥3 items, including dense infiltration of plasma cells and lymphocytes, storiform fibrosis, obliterative phlebitis, and abundant IgG4-positive cells (>10 cells per HPF). Level 2 requires two items, and Level 0 requires fewer than one item. Adverse events (AEs) were evaluated according to the lexicon for endoscopic AEs advocated by the American Society for Gastrointestinal Endoscopy workshop report [21].

### 2.5. Study Outcomes and Statistical Analysis

The primary outcome measure was comparing the diagnostic yield of Level 1 (≥3 items of ICDC) or Level 2 (2 items of ICDC) histological diagnostic criteria between 19FR and 22FR. Secondary outcome measures were adequate specimen rate, tissue area, correlation between tissue area and histological diagnostic yields, and AEs. Continuous variables are presented as medians and ranges. Intergroup comparisons were performed using the Mann-Whitney U test for continuous variables and the Fisher exact test for categorical variables. Spearman’s rank correlation coefficient was used to correlate tissue area with diagnostic performance. All tests were two-tailed, and a *p*-value of less than 0.05 was considered to indicate a statistically significant difference. R version 4.2.2 software was used for all statistical analyses.

## 3. Results

### 3.1. Patient’s Identification and Characteristics

**Figure 3:**
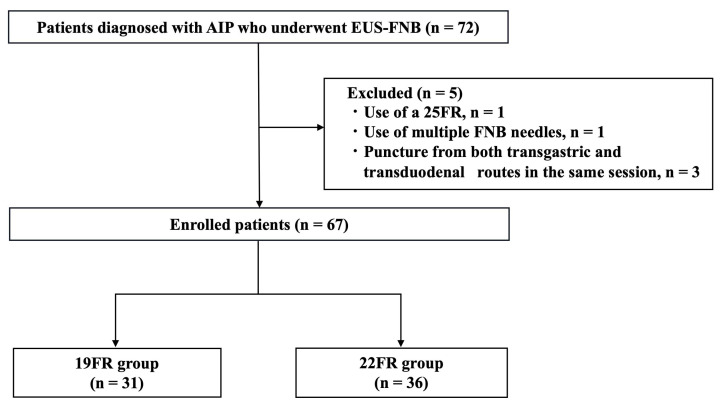
Flowchart of enrolled patients with autoimmune pancreatitis (AIP) who underwent ultrasound-guided fine-needle biopsy (EUS-FNB). FR, Franseen needle.

The database analysis identified 72 patients who underwent EUS-FNB using a Franseen needle and were ultimately diagnosed with AIP. However, five patients were excluded owing to the use of a 25FR (*n* = 1), the use of multiple FNB needles (*n* = 1), and puncture from both transgastric and transduodenal routes in the same session (*n* = 3). Ultimately, 67 patients who underwent EUS-FNB with a 19FR or 22FR and were ultimately diagnosed with AIP were included in this analysis. The 19FR group included 31 patients (46.3%), and the 22FR group included 36 patients (53.7%) (Figure 3). The baseline characteristics of the enrolled patients are shown in Table 1. There were no significant differences between the two groups in mean age, chief complaint, or area of pancreatic swelling, although the number of males tended to be higher in the 19FR group. The median serum IgG4 level was 368 mg/dL: 359 mg/dL in the 19FR group and 406 mg/dL in the 22FR group. A serum IgG4 level of ≥135 mg/dL accounted for more than 80% of patients in both groups (80.6% vs. 86.1%). Regarding other organ involvement, retroperitoneal fibrosis and IgG4-related sclerosing cholangitis were common. There were no differences between the two groups in the frequency of other organ involvement, presence of diabetes at diagnosis, or steroid use.

### 3.2. EUS-FNB Procedures

Regarding the route of puncture, transgastric puncture was performed in 30 (96.8%) of the 19FR group and 21 (58.3%) of the 22FR group, with transgastric puncture being significantly more common in the 19FR group (*p* < 0.001). The number of punctures was 2 (range: 1–5) in the 19FR group and 2 (range: 1–4) in the 22FR group (*p* = 0.543). Only one AE occurred in the 19FR group: minor bleeding that required endoscopic hemostasis (*p* = 0.460) (Table 2).

### 3.3. Comparison of Histological Outcomes Between 19FR and 22FR

The histological diagnoses of the enrolled patients are shown in Table 3. The adequate specimen rate was 100% (31/31) in the 19FR group and 97.2% (35/36) in the 22FR group. Regarding the presence of the four pathological features of AIP, lymphoplasmacytic infiltration was observed in 100% (31/31) of the 19FR group and 94.4% (34/36) of the 22FR group (*p* = 0.495). Storiform fibrosis was observed in 83.9% (26/31) of the 19FR group and 72.2% (26/36) of the 22FR group (*p* = 0.379). Immunostaining was used to evaluate IgG4-positive plasma cell infiltration in all cases. IgG4-positive plasma cells (>10 cells per HPF) were identified in 71.0% (22/31) of the 19FR group and 66.7% (24/36) of the 22FR group (*p* = 0.795). Notably, obliterative phlebitis was recognized at a significantly higher rate in the 19FR group (77.4%, 24/31) than in the 22FR group (38.9%, 14/36; *p* = 0.003). The primary outcome measure, the diagnostic rate based on Level 1 (≥3 items of ICDC), was observed to be higher in the 19FR group than in the 22FR group [90% (28/31) vs. 61.1% (22/36) (*p* = 0.010)]. The diagnostic rate of Level 2 (2 items of ICDC) showed no significant difference [9.7% (3/31) vs. 30.6% (11/36) (*p* = 0.068)]. The rates of Level 1 or Level 2 (≥2 items of ICDC) were not significantly different [100% (31/31) vs. 91.7% (33/36) (*p* = 0.243)], and the rates of Level 0 (≤1 item of ICDC) were not significantly different [0% (0/31) vs. 8.3% (3/36) (*p* = 0.243)] (Table 3).

### 3.4. Relationship of Histological Tissue Area and Histological Findings

**Figure 4:**
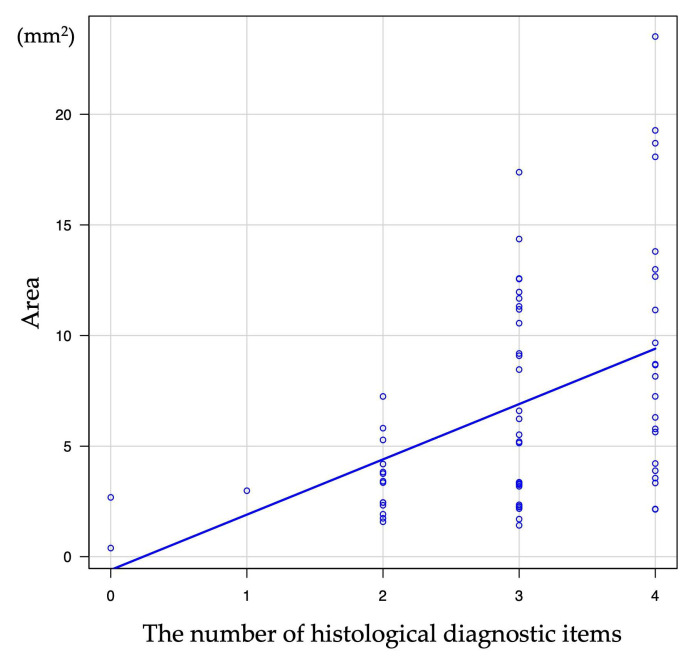
Correlation between tissue area and the number of diagnostic items.

**Figure 5:**
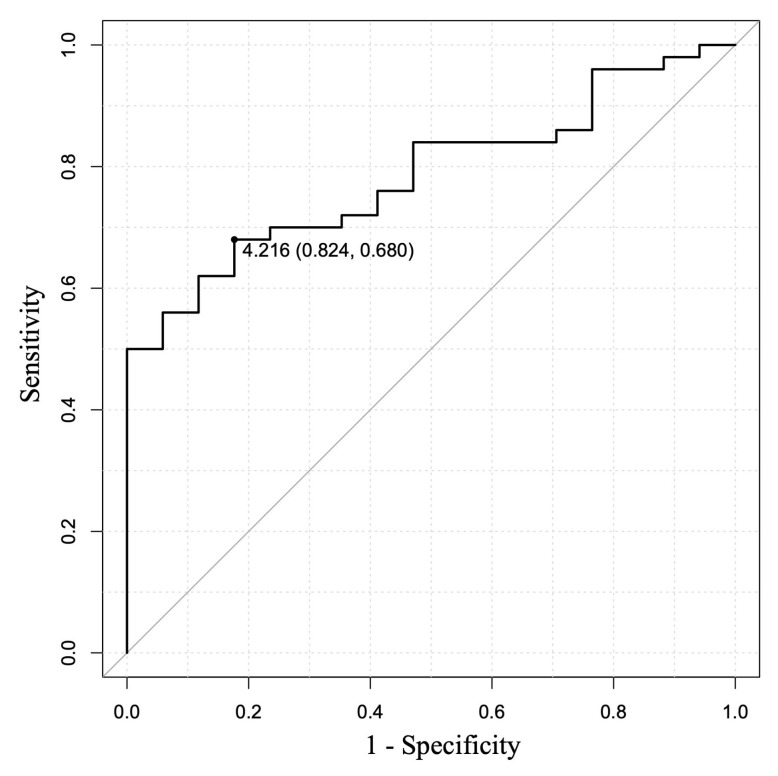
The receiver operating characteristic (ROC) curve for the tissue area is required to meet Level 1 (≥3 items of ICDC). The. Cutoff value of tissue area calculated based on Youden’s index was 4.22 mm^2^, with a sensitivity of 68.0%, specificity of 82.4%, and the area under the curve was 0.79 (95% confidence interval [CI], 0.68–0.89).

**Figure 6:**
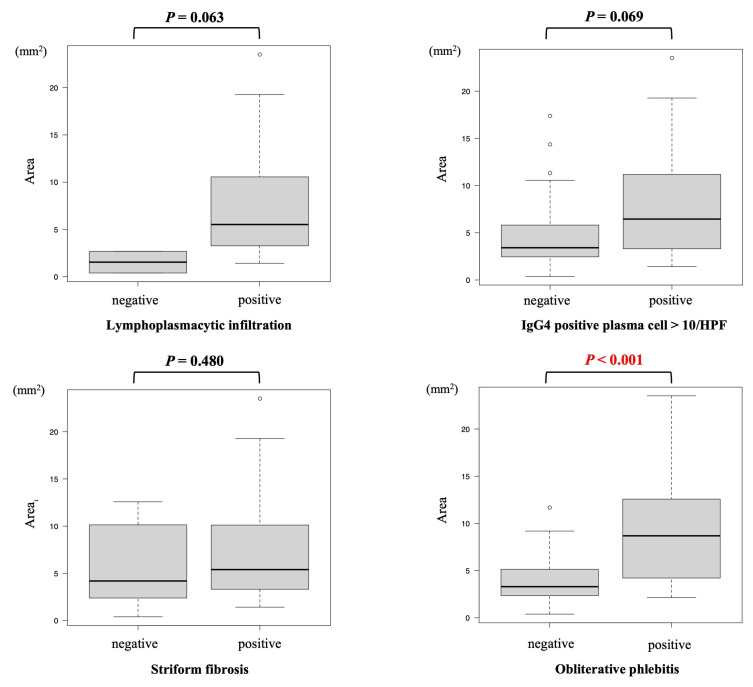
The association of each of the four histological diagnostic items with tissue area. HPF, high-power field.

The EUS-FNB outcomes and histological tissue areas are shown in Table 3. The 19FR group obtained significantly greater amounts of histological tissue samples compared to the 22FR group [9.19 mm^2^ (range: 2.14–23.51 mm^2^) vs. 3.36 mm^2^ (range: 0.39–12.58 mm^2^) (*p* < 0.001)]. In addition, the median histological tissue area per puncture was also significantly greater [4.82 mm^2^/pass (range: 0.72–12.67 mm^2^) vs. 1.83 mm^2^/pass (range: 0.20–12.56 mm^2^) (*p* < 0.001)]. The relationship between the number of histological diagnostic items and tissue area was 1.54 mm^2^ (range: 0.39–2.68 mm^2^) for 0 item, 2.99 mm^2^ for 1 item (in one case only), 3.38 mm^2^ (range: 1.58–7.25 mm^2^) for 2 items, 5.88 mm^2^ (range: 1.42–17.28 mm^2^) for 3 items, 8.41 mm^2^ (range: 2.16–23.51 mm^2^) for 4 items. The correlation coefficient was 0.47 (*p* < 0.001), indicating a positive correlation between tissue area and the number of histological diagnostic items (Table 4; Figure 4). To assess the tissue area required to meet Level 1 (≥3 items of ICDC), the receiver operating characteristic (ROC) curve was used. The cutoff value of the tissue area calculated based on Youden’s index was 4.22 mm^2^, with a sensitivity of 68.0%, specificity of 82.4%, and the area under the curve of 0.79 (95% confidence interval [CI], 0.68–0.89) (Figure 5). Considering the tissue area obtained per puncture, one appropriate puncture was required for the 19FR group and three appropriate punctures for the 22FR group to satisfy Level 1. Furthermore, the association of each of the four histological diagnostic parameters with tissue area showed that the area was significantly larger in patients with obliterative phlebitis (*p* < 0.001). No association with tissue area was observed for other histological diagnostic items, such as lymphoplasmacytic infiltration, IgG4-positive plasma cell infiltration, and storiform fibrosis (Table 5; Figure 6).

## 4. Discussion

This study primarily aimed to compare the histological diagnostic performance and tissue area of 19FR and 22FR for the diagnosis of AIP. The adequate specimen rate was 100% (31/31) in the 19FR group and 97.2% (35/36) in the 22FR group (*p* = 1.00). The diagnostic yield for Level 1 or Level 2 (≥2 items of ICDC) was 100% (31/31) in the 19FR group and 91.7% (33/36) in the 22FR group (*p* = 0.243). For Level 1 (≥3 items of ICDC), the diagnostic yields were 90.3% (28/31) in the 19FR group and 61.1% (22/36) in the 22FR group (*p* = 0.010). The detection rate of Level 1 was significantly higher in the 19FR group. With respect to tissue area, more specimens could be obtained in the 19FR group, and a larger area was required to fulfill a higher number of histological diagnostic items. The 19FR could achieve a Level 1 histological diagnosis with fewer punctures. Safety was similar in both groups.

As for the utility of FNB needles in comparison with FNA needles for the histological diagnosis of AIP, a recent meta-analysis revealed that FNB needles have a better diagnostic yield than FNA needles [22]. This meta-analysis, including nine studies with 309 patients for FNA needles and seven studies with 131 patients for FNB needles, showed that 60.1% (95% CI, 41.5–78.7%) with FNB needles and 30.0% (95% CI, 15.9–44.1%) (*p* = 0.008) with FNA needles met the Level 1 (≥3 items of ICDC) histology. In our study, the overall diagnostic performance of EUS-FNB, including both the 19FR and 22FR groups, showed that Level 1 (≥3 items of ICDC) was 74.6% (50/67) and Level 1 or Level 2 (≥2 items of ICDC) was 95.5% (64/67). FNB needles showed good performance for histological diagnosis, similar to the reported results of the meta-analysis. EUS-FNB is a useful method for reliably obtaining tissue samples from pancreatic lesions and enabling the histological diagnosis of AIP.

A variety of FNB needles have been invented, and FNB needles have different tip shapes and needle sizes, which could potentially affect the feasibility of the procedure and tissue acquisition capability. Concerning needle size, a subanalysis of a previous meta-analysis comparing FNA and FNB needles in the diagnosis of AIP showed a higher diagnostic yield with 19-gauge needles than with 22-gauge needles, although the results included both FNA and FNB needles. However, few studies have directly compared the histological diagnostic performance of 19-gauge and 22-gauge FNB needles for AIP. Our study compared histological diagnostic yields of AIP between 19FR and 22FR and showed no difference in histological diagnostic yield of ICDC Level 1 or Level 2 (≥2 items of ICDC) between the two groups, 100% (31/31) vs. 91.7% (33/36) (*p* = 0.243). On the other hand, the detection rate of Level 1 (≥3 items of ICDC) in the 19FR group was significantly higher than that in the 22FR group (90.3% vs. 61.1%, *p* = 0.010), and the 19FR group showed a significantly higher rate of definitive diagnosis of AIP. Ishikawa et al. [23] prospectively evaluated the histological diagnostic performance of EUS-FNB using 19FR for 20 patients with AIP and compared it with a historical control group using 22FR for 29 patients with AIP. The results showed no significant difference in the histological diagnostic yields for Level 1 or 2 (≥2 items of ICDC: 80.0% vs. 72.4%; *p* = 0.398), but a better tendency for Level 1 (≥3 items of ICDC: 65.0% vs. 41.3%; *p* = 0.104). Based on these two study results, the application of a large-caliber FNB needle could improve the histological diagnostic yields for AIP. As for the sampling technique for pancreatic lesions, Crinò SF et al. showed that the wet-suction technique in EUS-FNB obtains significantly more histological tissue samples. The technique may also improve the diagnostic performance of definitive diagnosis of AIP [24,25].

Regarding the histological tissue area in EUS-FNB for AIP diagnosis, our study demonstrated that the tissue area obtained with EUS-FNB for pancreatic lesions was significantly larger in the 19FR group than in the 22FR group. Per session analysis revealed 9.19 mm^2^/session vs. 3.36 mm^2^/session (*p* < 0.001), respectively. The median tissue area per puncture was also significantly larger in the 19FR group than in the 22FR group (4.82 mm^2^/pass vs. 1.83 mm^2^/pass; *p* < 0.001). These findings indicate that both the total and median tissue areas obtained per puncture were significantly larger with the 19FR. Ishikawa et al. reported similar findings, with 19FR yielding a significantly larger specimen than the 22FR (median: 11.9 mm^2^ vs. 8 mm^2^; *p* = 0.027) [23]. Collectively, these reports suggest that 19FR provides a larger tissue area than 22FR.

In the analysis of the histological area and diagnosis of AIP, our study showed a positive correlation between the tissue area and the number of histological diagnostic items of AIP. The ideal cutoff value of tissue area to achieve Level 1 histological diagnosis (≥3 items of ICDC) was 4.22 mm^2^. The median tissue area for a single puncture in the 19FR group was 4.82 mm^2^. Therefore, if an appropriate specimen is obtained by EUS-FNB using 19FR, a single puncture may be sufficient to meet the Level 1 histological diagnosis. Conversely, the median tissue area per puncture for the 22FR group was 1.83 mm^2^, necessitating three appropriate punctures to meet Level 1 histological diagnosis. This highlights the potential of 19-gauge FNB needles to obtain Level 1 histological diagnosis with fewer punctures. However, it is noteworthy that the sensitivity remains relatively low, even with the use of FR. We should keep in mind the limitations of histological diagnosis with the use of EUS-FNB, and comprehensive diagnosis is needed, considering imaging features and serology.

When assessing the relationship between the four histological diagnostic items of ICDC and tissue area, obliterative phlebitis had the lowest detection rate among the four histological findings: 77.4% (24/31) in the 19FR group and 38.9% (14/36) in the 22FR group. Obliterative phlebitis is often challenging to identify, with previous reports showing detection rates of 0–44% using EUS-FNA/FNB [8,9,10,13,26,27,28,29]. Our study demonstrated a significantly higher detection rate of obliterative phlebitis in the 19FR group than in the 22FR group (77.4% vs. 38.9%; *p* = 0.003). This discrepancy may be attributed to variations in the use of elastic stains, such as EVG staining, which has been shown to improve the detection of obliterative phlebitis [30]. In our analysis of tissue area and individual histological diagnostic items, the tissue area was significantly larger in cases where obliterative phlebitis was present. This suggests that a larger tissue specimen may be necessary to evaluate obliterative phlebitis, further supporting the use of 19FR.

Our results suggest that using 19FR for AIP diagnosis improves diagnostic performance by obtaining a larger tissue specimen with fewer punctures compared to 22FR. However, large-diameter needles have limitations in endoscopic manipulation owing to increased rigidity and resistance during puncture. This may reduce the feasibility of EUS-FNA in cases that require an angulated scope position, such as transduodenal punctures. Our study observed fewer transduodenal punctures in the 19FR group than in the 22FR group. It is reasonable to consider that endoscopists encountered more difficulty using large-diameter needles through the duodenum, and this point could have impacted the area of the obtained tissue sample. When selecting a puncture needle for AIP diagnosis, particularly in cases requiring an angulated scope position, 19-gauge needles made of Nitinol, which offer lower needle stiffness, or 22-gauge needles should be considered.

Kanno et al. reported a 1.7% incidence of EUS-FNA-related AEs in a multicenter retrospective study of 13,566 cases [31]. Bleeding was the most frequent AE; however, no fatalities occurred, indicating the relative safety of the procedure. While the frequency of EUS-FNA-related AEs tended to be slightly higher for AIP (2.1%) than for pancreatic cancer (1.6%), our study found only one case (3.2%) of bleeding in the 19FR group, with no difference in the frequency of bleeding in the 22FR group (0%; *p* = 1.00). Ishikawa et al. also reported no difference in AE frequency between the 19FR and 22FR used for AIP diagnosis [23]. These results indicate that the use of 19-gauge needles, which can obtain larger tissue specimens, is safe and desirable, provided that puncture difficulty is not increased owing to scope angulation.

This study had several limitations, including its retrospective design, small sample size, and lack of complete protocol standardization. According to the puncture site, we examined cases in which the puncture site was limited to either the stomach or the duodenum. It should be noted that the results are not applicable to complex cases requiring puncture from multiple sites. In this study, we focused primarily on the histological diagnostic capability of EUS-FNB for AIP, so the cost-effectiveness analysis has not been performed in the selection of the puncture needle. These factors may have introduced bias and influenced the diagnostic performance. Additionally, histological evaluation was performed by a single expert pathologist at our hospital, potentially limiting external validity.

## 5. Conclusions

In conclusion, the EUS-FNB performed with 19FR allows for the acquisition of larger tissue specimens with fewer punctures than 22FR in the histological diagnosis of AIP. Larger tissue samples may enable a definitive diagnosis of AIP. Prospective comparative studies are needed to further evaluate the feasibility and diagnostic performance of 19FR.

## Figures and Tables

**Table 1:** Baseline characteristics of patients (*n* = 67).

	19 FR	22 FR	*p*-Value
	(*n* = 31)	(*n* = 36)	
Sex, male, *n* (%)	29 (93.5)	26 (72.2)	0.028
Age, yo, median (range)	74 (21–87)	69 (45–80)	0.365
Chief complaint, *n* (%)			0.139
Pancreatic enlargement	23 (74.2)	16 (44.4)	
Jaundice or liver dysfunction	6 (19.4)	13 (36.1)	
Abdominal pain	2 (6.5)	4 (11.1)	
Others	0	3 (8.3)	
Pancreatic enlargement, *n* (%)			0.243
Diffuse (≥2/3)	11 (35.5)	16 (44.4)	
Segmental (1/3–2/3)	10 (32.3)	5 (13.9)	
Focal (<1/3)	10 (32.3)	15 (41.7)	
Capsule-like rim, *n* (%)	20 (64.5)	14 (38.9)	0.051
Serum IgG4, mg/dL, median (range)	359 (8–2710)	406 (75.1–1690)	0.930
IgG4 ≥ 135 mg/dL, *n* (%)	25 (80.6)	31 (86.1)	0.742
IgG4 ≥ 270 mg/dL, *n* (%)	20 (64.5)	23 (63.9)	1.000
Other organ involvement, *n* (%)			
Sclerosing cholangitis	6 (19.4)	10 (27.8)	0.567
Retroperitoneal fibrosis	9 (29)	7 (19.4)	0.401
IgG4-related respiratory disease	0	2 (5.6)	0.495
Sialadenitis	0	2 (5.6)	0.495
Dacryoadenitis	0	2 (5.6)	0.495
Hypophysitis	1 (3.2)	1 (2.8)	1.000
Ulcerative colitis	1 (3.2)	0	1.000
IgG4-related kidney disease	0	1 (2.8)	1.000
Diabetes Mellitus, *n* (%)	12 (38.7)	17 (47.2)	0.622
Treatment, *n* (%)			
Steroid administration	17 (54.8)	26 (72.2)	0.202

**Table 2:** Characteristics of the EUS-FNB procedure.

	19 FR	22 FR	*p*-Value
	(*n* = 31)	(*n* = 36)	
Route for puncture, *n* (%)			<0.001
Transgastric	30 (96.8)	21 (58.3)	
Transduodenal	1 (3.2)	15 (41.7)	
Number of passes, median (range)	2 (1–5)	2 (1–4)	0.543
Adverse events, *n* (%)			
Bleeding	1 (3.2)	0	0.460

EUS-FNB, endoscopic ultrasound-guided fine needle biopsy.

**Table 3:** Histological findings and acquired tissue area.

	19 FR	22 FR	*p*-Value
	(*n* = 31)	(*n* = 36)	
Specimen adequacy, *n* (%)	31 (100)	35 (97.2)	1.000
Pathological findings, *n* (%)			
Lymphoplasmacytic infiltration	31 (100)	34 (94.4)	0.495
Storiform fibrosis	26 (83.9)	26 (72.2)	0.379
Obliterative phlebitis	24 (77.4)	14 (38.9)	0.003
IgG4 positive plasma cells > 10/HPF	22 (71.0)	24 (66.7)	0.795
ICDC diagnostic level, *n* (%)			
Level 0 (items ≤ 1)	0	3 (8.3)	0.243
Level 1 (items ≥ 3)	28 (90.3)	22 (61.1)	0.010
Level 2 (2 items)	3 (9.7)	11 (30.6)	0.068
Level 1 or 2 (items ≥ 2)	31 (100)	33 (91.7)	0.243
Tissue area, mm^2^/session, median (range)	9.19 (2.14–23.51)	3.36 (0.39–12.58)	<0.001
Tissue area, mm^2^/pass, median (range)	4.82 (0.72–12.67)	1.83 (0.20–12.56)	<0.001

FR, Franseen needle; ICDC, International Consensus Diagnostic Criteria; HPF, high-power field.

**Table 4:** The relationship between the number of histological diagnostic items and tissue area.

Items of Histological Findings, *n* (%)		Tissue Area, mm^2^, Median (Range)
0	2 (3.0)	1.54 (0.39–2.68)
1	1 (1.5)	2.99
2	14 (20.9)	3.38 (1.58–7.25)
3	28 (41.8)	5.88 (1.42–17.28)
4	22 (32.8)	8.41 (2.16–23.51)

**Table 5:** Relationship between acquired tissue area and histological findings.

	Histological Finding	Tissue Area, mm^2^, Median, (Range)		*p*-Value
Lymphoplasmacytic infiltration	positive	5.52 (1.42–23.51)	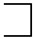	0.063
	negative	1.54 (0.39–2.68)		
Storiform fibrosis	positive	5.40 (1.42–23.51)	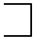	0.480
	negative	4.19 (0.39–12.58)		
Obliterative phlebitis	positive	8.69 (2.14–23.51)	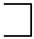	<0.001
	negative	3.30 (0.39–11.68)		
IgG4 positive plasma cell > 10/HPF	positive	6.45 (1.42–23.51)	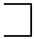	0.069
	negative	3.42 (0.39–17.38)		

## Data Availability

The data supporting the reported results of this study are not publicly available due to privacy and ethical restrictions. The authors make data available upon request.

## Abbreviations

The following abbreviations are used in this manuscript:

AIP	Autoimmune pancreatitis
EUS-FNA	Endoscopic ultrasound–guided fine needle aspiration
EUS-FNB	Endoscopic ultrasound–guided fine needle biopsy
EVG	Elastica van Gieson
FR	Franseen needle
HE	Hematoxylin and eosin
HPF	High-power field
ICDC	International Consensus Diagnostic Criteria
IDCP	Idiopathic duct-centric pancreatitis
LPSP	Lymphoplasmacytic sclerosing pancreatitis
OOI	Other organ involvement
ROC	Receiver operating characteristic
